# The effect of goal-directed fluid therapy on endothelial glycocalyx and short-term postoperative recovery quality in older patients undergoing laparoscopic hernia or gallbladder surgery: a randomized controlled double-blind clinical study

**DOI:** 10.3389/fmed.2026.1922109

**Published:** 2026-10-14

**Authors:** Qinxian Wei, Man Zhou, Wenji Xu, Jiaxin Wan, Wuchang Fu, Fangjun Wang

**Affiliations:** 1 Affiliated Hospital, North Sichuan Medical College, Nanchong, China; 2 Second Clinical Medical Institution of North Sichuan Medical College (Nanchong Central Hospital), Nanchong, China

**Keywords:** endothelial glycocalyx, goal-directed fluid therapy, inflammation, older patients, postoperative recovery quality

## Abstract

**Background:**

This study aimed to observe the effects of intraoperative goal-directed fluid therapy on postoperative recovery in older patients undergoing laparoscopic hernia or gallbladder surgery.

**Methods:**

A total of 60 older patients were randomly assigned to the control group (C group) and the goal- directed fluid therapy group (G group). The primary outcome was the plasma concentration of syndecan-1. The secondary outcomes were the plasma concentrations of tumor necrosis factor alpha (TNF-α), interleukin-1 (IL-1), intercellular adhesion molecule-1 (ICAM-1), and vascular cell adhesion molecule-1 (VCAM-1); mean arterial pressure; heart rate; regional cerebral oxygen saturation; intraoperative dopamine dosage; QoR-15 scale score; hematocrit; and postoperative adverse events.

**Results:**

Compared with the C group, the G group showed decreased plasma concentrations of ICAM-1 at the end of surgery and 24 h after surgery (*P* = 0.010 and 0.005, respectively), decreased plasma concentrations of syndecan-1 at the end of surgery (*P* = 0.049), more significantly decreased plasma concentrations of VCAM-1 at the end of surgery and 24 h after surgery (*P* < 0.001 and *P* = 0.017, respectively), and decreased plasma concentrations of IL-1β at the end of surgery and 24 h after surgery (*P* = 0.009 and 0.008, respectively). The C group showed increased plasma concentration of TNF-α at the end of surgery and 24 h after surgery compared with those before surgery (*P* = 0.003 and 0.023, respectively). Compared with the C group, the QoR-15 scores were higher at 24, 48, and 72 h after surgery (*P* = 0.001, 0.001, and 0.005, respectively) in the treatment group.

**Conclusion:**

Intraoperative goal-directed fluid therapy in non-critically ill older patients may alleviate endothelial glycocalyx degradation with a lower incidence of adverse events.

**Clinical Trial Registration:**

http://www.chictr.org.cn/, Chinese Clinical Trial Registry ChiCTR2500097999.

## Background

The endothelial glycocalyx (EG) is located between the blood flow and the vascular endothelium and is a crucial determinant of vascular permeability. It is rich in carbohydrates and is interconnected by glycosaminoglycans and soluble proteoglycans, maintaining a dynamic balance with the flowing blood and continuously affecting the thickness of the glycocalyx (1). Endothelial glycocalyx degradation can lead to increased vascular permeability, organ dysfunction, increased inflammatory response, and coagulation imbalance (2). The endothelial glycocalyx deteriorates with age, and it becomes particularly fragile in older patients (3).

Common causes of hypotension during surgery include insufficient blood volume, excessive anesthesia, cardiac dysfunction, and vasodilation, which require fluid replacement, use of vasopressors, and adjustment of anesthesia depth for intervention. Inappropriate fluid infusion during surgery may cause damage to EG, leading to its detachment, increased vascular permeability, inflammatory response, and postoperative complications, resulting in poor prognosis of surgical patients (4). Previous studies have found that goal-directed fluid therapy (GDFT) in critically ill patients can effectively protect the endothelial glycocalyx and improve the prognosis of patients (5). Therefore, we hypothesized that goal-directed fluid therapy would protect the endothelial glycocalyx and improve postoperative recovery in older patients.

However, it is unclear whether intraoperative goal-directed fluid therapy can improve postoperative recovery in non-critically ill older patients. We developed this study to observe the effects of intraoperative goal-directed fluid therapy on postoperative recovery in older patients undergoing laparoscopic hernia or gallbladder surgery.

## Methods

This clinical trial was approved by the Medical Ethics Committee of Nanchong Central Hospital of Sichuan province (ethical approval no.: 2024 audit no. [181]) and was registered at the Chinese Clinical Trial Registry (http://www.chictr.org.cn/; registration number: ChiCTR2500097999; registration date: 28 February 2025). Between March 2025 and December 2025, patients aged ≥75 years with American Society of Anesthesiologists (ASA) grades II and III and body mass index (BMI) = 18.5–30 kg/m^2^ who were scheduled for laparoscopic cholecystectomy or hernia repair surgery were enrolled. Signed informed consent forms were obtained from the patients and their families prior to the study. This clinical study adheres to the Declaration of Helsinki and Consolidated Standards of Reporting Trials (CONSORT) guidelines (6). Exclusion criteria were diabetes or impaired glucose tolerance, severe cardiovascular disease (New York Heart Association (NYHA) grade III or above), poor control of hypertension (blood pressure above 160/90 mmHg or driven by irregular medication), cerebrovascular disease, a weight loss of more than 10% over the past 6 months, pyloric obstruction, gastroesophageal reflux, serious liver and kidney disease, hypoproteinemia, alcohol and drug abuse, anemia, and central nervous system dysfunction. Withdrawal standards were intraoperative blood loss ≥500 mL, incomplete data collection, change of surgical plan, and postoperative loss to follow-up.

Older patients were randomly divided into the control group (C group) and goal-directed fluid therapy group (G group), with 30 cases in each group. Randomization was carried out by an anesthesiologist who was not involved in this study using a random number table method. Sixty 2-digit numbers were randomly selected from the random number table and arranged in ascending order; then, serial numbers 1–30 were set for the control group, and 31–60 were set for the goal-directed fluid therapy group. The grouping information was written on the card and sealed in an opaque envelope. The surgeons, older patients, outcomes assessor, operating room and ward nurses, and statistical analysts were blinded to the grouping. After the patients arrived at the operation room, a sealed envelope was sequentially drawn, and the intraoperative fluid therapy strategy for the patients according to the information on the card in the sealed envelope was implemented by the anesthesiologist who is in charge of patient anesthesia and who was not involved in the assessment of outcomes and statistical analysis. The primary outcome was the plasma concentrations of syndecan-1 at the end of surgery. The secondary outcomes were the plasma concentrations of syndecan-1 (30 min before surgery and 24 h after surgery), tumor necrosis factor alpha (TNF-α), interleukin-1 (IL-1), intercellular adhesion molecule-1 (ICAM-1), and vascular cell adhesion molecule-1 (VCAM-1); mean arterial pressure (MAP); heart rate (HR); regional cerebral oxygen saturation (rSO_2_); intraoperative dopamine dosage; QoR-15 scale score; hematocrit (HCT); and postoperative adverse events.

All older patients without premedication were asked to fast for 8 h and forbidden to drink for 4 h before surgery. After the patients entered the operating room, an upper limb venous channel was established for them, followed by intravenous administration of 8 mL/kg·h of Ringer's solution. Non-invasive blood pressure (NIBP), electrocardiogram (ECG), and spontaneous pulse oximetry (SpO_2_) were routinely monitored. After successful catheterization of the left radial artery under local anesthesia, the patients were administered 1.0–2.0 mg/kg of propofol, 0.3 μg/kg of sufentanil, and 0.2 mg/kg of etomidate for anesthesia induction via intravenous injection. All patients underwent mechanically volume-controlled ventilation with a tidal volume of 8–10 mL/kg, a respiratory rate of 10–14 breaths/min, a positive end-expiratory pressure of 5 cmH_2_O, and an end-tidal carbon dioxide partial pressure of 35–45 mmHg after tracheal intubation. Intravenous infusion of 4–8 mg/kg·h of propofol and 0.1–0.2 μg/kg of remifentanil was performed to maintain a bispectral index (BIS) value of 45–60 during surgery. Intravenous infusion of 2 mg of cisatracurium was administered intermittently as needed. In the goal-directed fluid therapy group, the FloTrac/Vigileo system was used to monitor intraoperative indicators such as variability of stroke volume (SVV), cardiac index (CI), and stroke volume index (SVI). When CI ≥2.5 L/(min·m^2^), MAP >65 mmHg, and SVI >35 mL/m^2^, no treatment measures were required. If CI < 2.5 L/(min·m^2^), MAP <65 mmHg, SVI <35 mL/m^2^, and SVV <13%, after intravenous infusion of 250 mL of hydroxyethyl starch 130/0.4 (30 g of hydroxyethyl starch 130/0.4 with 4.5 g of sodium chloride), 5 μg/(kg·min) of dopamine was administered. If SVV >13%, 3–5 mL/kg of Ringer's solution was administered rapidly, and re-evaluation was performed after 5 min. If SVV remained greater than 13%, 250 mL of hydroxyethyl starch 130/0.4 was administered. If CI <2.5 L/(min·m^2^) and SVI >35 mL/m^2^, 5 μg/(kg·min) of dopamine was administered via intravenous infusion. In the control group, Ringer's solution and hydroxyethyl starch 130/0.4 were administered via intravenous infusion to replenish the intraoperative blood loss. The ratio of hydroxyethyl starch 130/0.4 to blood loss was 1:1 (mL). For patients with continuous intraoperative hypotension, dopamine 2–10 μg (kg·min) was intravenously administered to maintain MAP > 65 mmHg.

Oxycodone (5 mg) and tropisetron (5 mg) were administered intravenously 30 min before the end of surgery. The administration of intravenous infusion of remifentanil and propofol was stopped at the end of the surgery. Older patients were transferred to the post-anesthesia care unit (PACU) for vital signs monitoring and continuation of supplemental oxygen supply through a mask after extubation. If the Steward score was ≥4, the patient was sent back to the ward.

Venous blood (5 mL) was collected 30 min before surgery (T_0_), at the end of surgery (T_1_), and 24 h after surgery (T_2_). The samples were centrifuged at 1,000 × *g* for 15 min, and then, the plasma samples were stored at −20 °C for the detection of plasma concentrations of syndecan-1, TNF-α, IL-1, ICAM-1, and VCAM-1 using the enzyme-linked immunosorbent assay with Syndecan-1, TNF-α, IL-1, ICAM-1, and VCAM-1 assay kits ZCIBIO Technology Co., Ltd., Shanghai, China. The MAP, HR, and rSO_2_ were recorded 5 min before anesthesia (t_1_), immediately after induction (t_2_), 30 min after pneumoperitoneum (t_3_), and the end of surgery (t_4_). The HCT was measured 30 min before surgery, at the end of surgery, and 3 h after surgery. QoR-15 scale scoring was performed on the day before surgery and at 24, 48, and 72 h after surgery. The outcomes were assessed by a trained anesthetic nurse who was blinded to the grouping.

## Statistical analysis

PASS15.0 software was used to calculate the sample size. A pilot experiment showed that the plasma concentrations of syndecan-1 at the end of surgery in the control group and the goal-directed fluid therapy group were 49.35 ± 12.6, and 38.2 ± 11.3, respectively. With the probability of type 1 error set at 5% and with 90% statistical power, 24 subjects were required per group. To account for a 20% dropout rate, 60 older patients were recruited in this study. The sample size was calculated by using the following formula:

$$n=\frac{{\left(Z_{\alpha }+Z_{\beta }\right)}^{2}\times 2\sigma^{2}}{\delta^{2}}$$

SPSS 21.0 software was used to perform the statistical analysis. The measurement data with normal distribution were expressed as mean ± standard deviation (SD), the measurement data with non-normal distribution were represented as the median (P_25_, P_75_), and the count data were presented as frequencies. The mean differences between groups for weight, height, age, surgery time, and duration of postoperative hospitalization were compared by using one-way analysis of variance (ANOVA), and for the gender ratio, the ASA physical status, types of surgery, and the incidence of adverse event were compared by using a chi-square test or Fisher's exact test. The serum concentrations of syndecan-1, IL-1β, ICAM-1, TNF-α, and VCAM-1 between groups at different time points were compared by using repeated-measures analysis of variance. If the comparison between groups was positive, the Student–Newman–Keuls (SNK) *post-hoc* test was performed with Bonferroni correction. A *P-*value of <0.05 was considered statistically significant.

## Results

A total of 72 older patients were assessed for eligibility in this study. Six patients who had diabetes, three patients with cardiovascular and cerebrovascular diseases, one patient with serious liver and kidney disease, and two patients with hypoproteinemia were excluded. The remaining 60 older patients were subsequently assigned to two groups, with 30 cases for each group. No patients were withdrawn from this clinical trial. All 60 patients completed this study (Figure 1).

**Figure 1:**
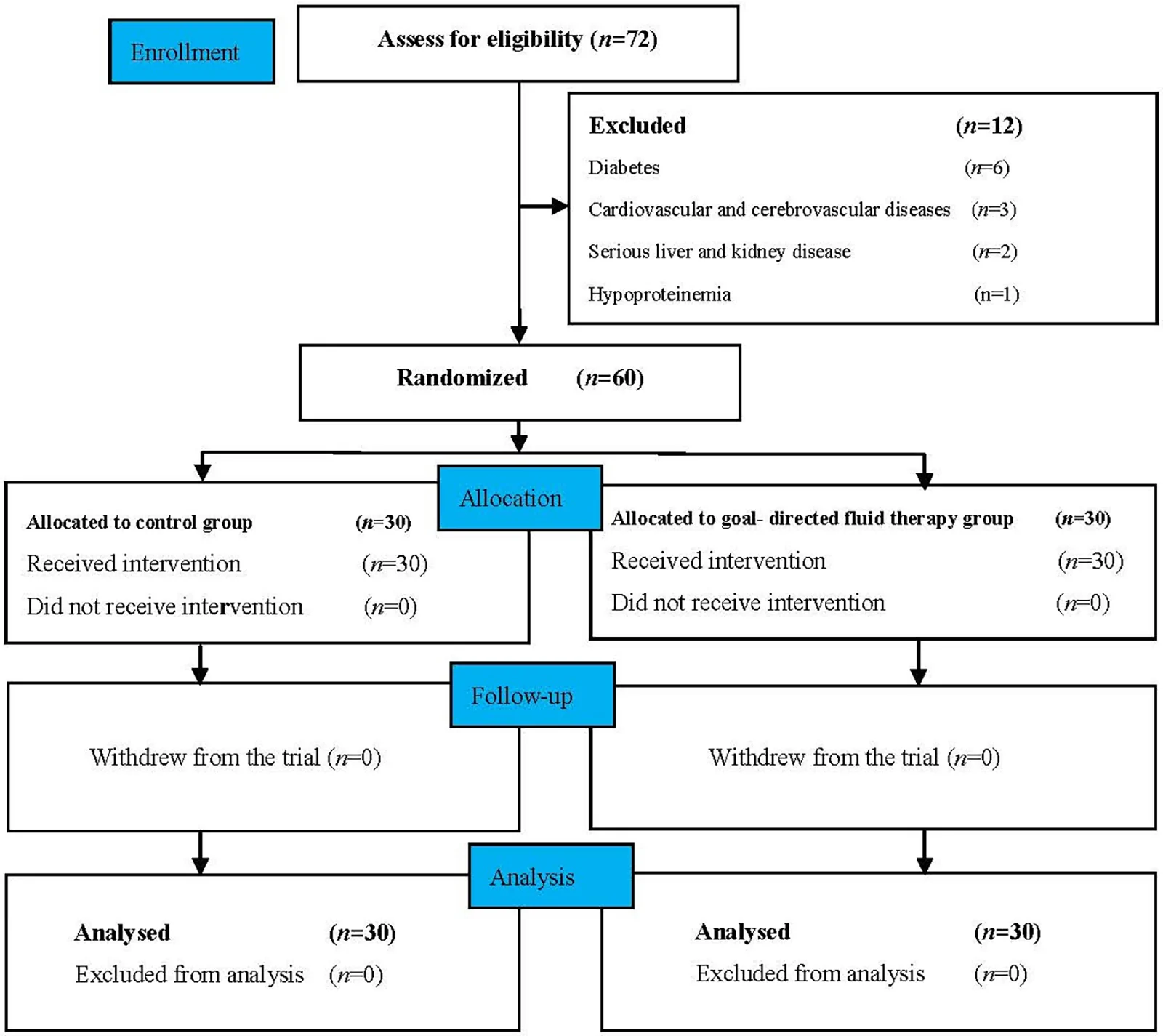
A participant flow diagram.

The demographic data are presented in Table 1. There was no difference between the two groups in terms of weight, height, age, gender, ASA classification, types of surgery, surgery time, and duration of hospitalization (*P* = 0.673, 0.256, 0.539, 0.519, 0.592, 0.605, 0.394, and 0.063. respectively), as seen in Table 1.

**Table 1:** Demographic data.

Characteristic	C group	G group	*t*/*χ^2^* values	*P*-values
Weight, mean ± SD (kg)	63.2 ± 6.0	62.6 ± 5.5	0.424	0.673
Height, mean ± SD (cm)	163.7 ± 6.2	165.6 ± 6.6	1.147	0.256
Age, mean ± SD (year)	79.9 ± 3.4	80.6 ± 4.8	0.618	0.539
Gender, *n* (%)			0.417	0.519
Male	23 (76.7)	25 (83.3)		
Female	7 (23.3)	5 (16.7)		
ASA, *n* (%)			0.287	0.592
II	20 (66.7)	18 (60)		
III	10 (33.3)	12 (40)		
Types of surgery, *n* (%)			0.268	0.605
Laparoscopic inguinal hernioplasty	15 (50)	17 (56.7)		
Laparoscopic cholecystectomy	15 (50)	13 (43.3)		
Surgery time, mean ± SD (min)	50.6 ± 4.8	51.8 ± 5.9	0.858	0.394
Duration of hospitalization, mean ± SD (days)	3.7 ± 1.1	3.2 ± 1.0	1.897	0.063

C group, control group; G group, goal-directed fluid therapy group; ASA, American Society of Anesthesiologists.

Values are expressed as mean ± SD and the number of patients. Differences in weight, height, age, surgery time, and duration of hospitalization between the two groups were analyzed using an independent samples t-test, and those in gender, ASA class, and types of surgery between the groups were analyzed using a chi-square test.

The plasma concentrations of ICAM-1, IL-1β, syndecan-1, TNF-α, and VCAM-1 in the two groups are shown in Figure 2. The plasma concentration data of ICAM-1, IL-1β, syndecan-1, TNF-α, and VCAM-1 did not violate sphericity. The two-way repeated-measures analysis of variance for the plasma concentration data of ICAM-1, IL-1β, syndecan-1, TNF-α, and VCAM-1 showed that the time effect of ICAM-1, IL-1β, syndecan-1, TNF-α, and VCAM-1 were significant in both the groups (all *P* < 0.001), the time × group effect of ICAM-1, IL-1β, and VCAM-1 were significant in both the groups (*P* = 0.047, 0.006, and <0.001, respectively), and the group effect of ICAM-1, IL-1β, and VCAM-1 was significant in the two groups (*P* = 0.021, 0.040, and 0.016, respectively). The plasma concentration of ICAM-1 was significantly higher at the end of surgery than at the day before surgery (*P* = 0.006). Compared with the C group, the plasma concentration of ICAM-1 decreased more significantly at the end of surgery and at 24 h after surgery in the G group (*P* = 0.010 and 0.005, respectively). The plasma concentrations of IL-1β in the C group were significantly higher at the end of surgery and at 24 h after surgery than those in the G group (*P* = 0.009 and 0.008, respectively). Compared with the plasma concentration of IL-1β before surgery, the plasma concentration of IL-1β was high at the end of surgery and at 24 h after surgery in the C group (*P* < 0.001 and *P* = 0.001, respectively) and at the end of surgery in the G group (*P* = 0.040). There was no difference in the plasma concentration of syndecan-1 between time points in the G group (*P* = 0.492). The plasma concentration of syndecan-1 in the C group was significantly higher at the end of surgery than that before surgery (*P* = 0.006), and it returned to the preoperative level 1 day after surgery (*P* = 0.735). The plasma concentration of syndecan-1 in the G group was significantly higher at the end of surgery (*P* = 0.049) than in the C group. The plasma concentration of TNF-α in the C group was significantly higher at the end of surgery and at 24 h after surgery than that before surgery (*P* = 0.003 and 0.023, respectively). The plasma concentration of VCAM-1 was higher at the end of surgery and at 24 h after surgery in the C group (all *P* < 0.001) and at the end of surgery in the G group (*P* < 0.001) than that before surgery. The plasma concentration of VCAM-1 was significantly lower at the end of surgery and at 24 h after surgery in the G group than that in the C group (*P* < 0.001 and *P* = 0.017, respectively).

**Figure 2:**
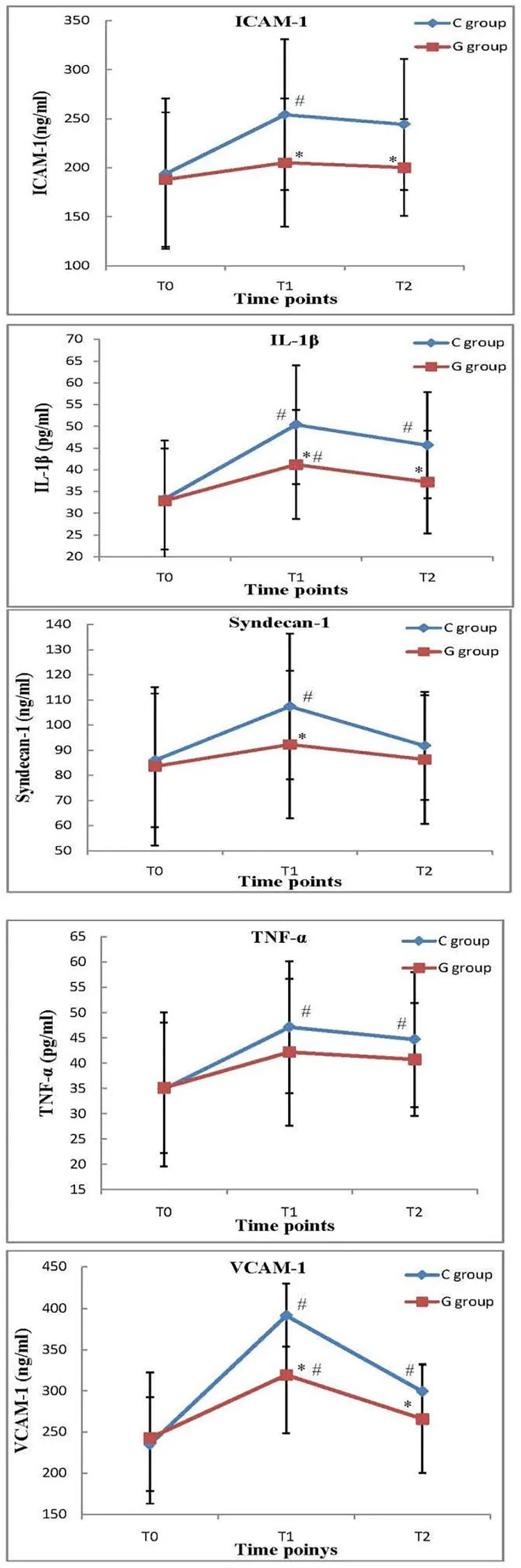
The plasma concentrations of ICAM-1, IL-1β, syndecan-1, TNF-α, and VCAM-1 in the two groups (*P < 0.05) vs*.* the C group (^#^*p* < 0.017) vs*.* T_0_.

The MAP, HR, rSO_2_, and HCT at different time points in the two groups are shown in Figure 3. Because the MAP, HR, rSO_2_, and HCT data violated sphericity, a two-way repeated-measures ANOVA with Greenhouse–Geisser correction was used. The time effects of MAP, HR, rSO_2_, and HCT were significant in the two groups (*P* < 0.001, *P* < 0.001, *P* = 0.003, and *P* < 0.001, respectively), the time × group effects of rSO_2_ and HCT were significant in the two groups (*P* = 0.028 and *P* < 0.001, respectively), and the group effects of rSO_2_ were significant in the three groups (*P* = 0.008). The MAP was lower at t_2_ (all *P* < 0.001) and higher at t_4_ in both groups (*P* < 0.001 and *P* = 0.004, respectively) after surgery than those before surgery. The HR was significantly lower at t_2_ in both groups after surgery (*P* < 0.001 and *P* = 0.005, respectively) than that before surgery. There was no difference in the HR between groups at different time points (*P* > 0.05). The HCT was significantly lower at t_4_ and t_5_ in the C group (all *P* < 0.001).

**Figure 3:**
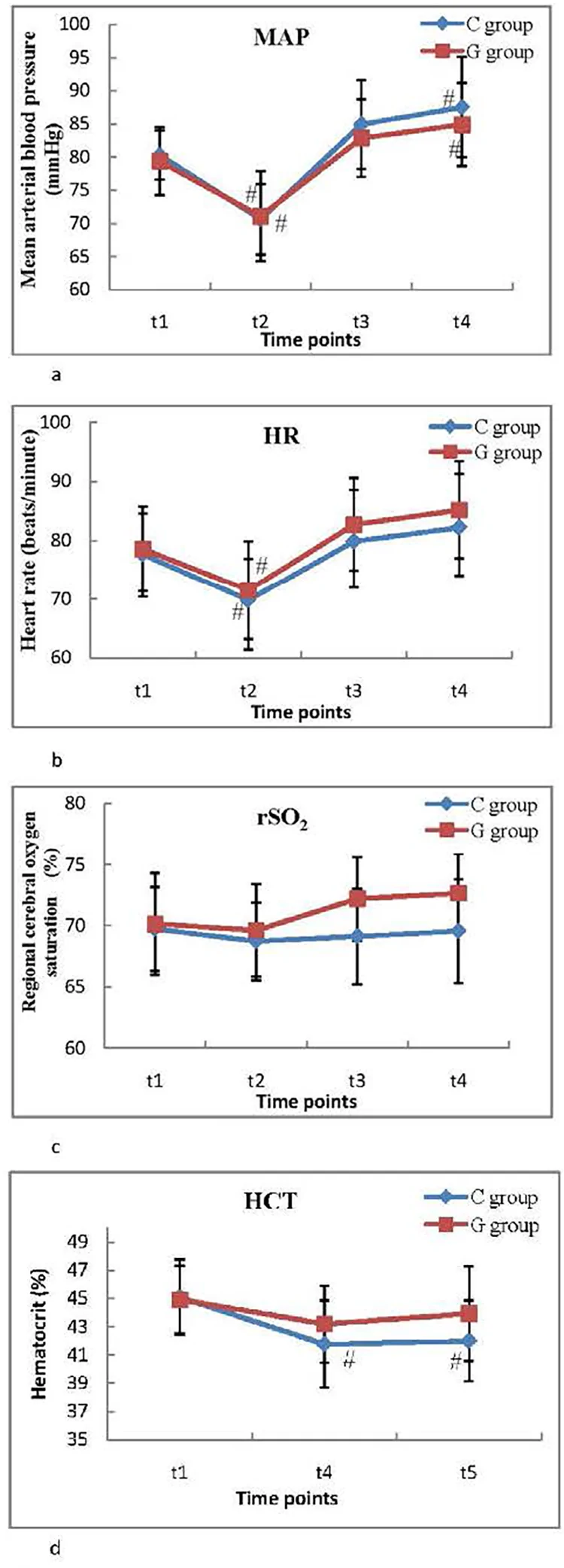
The MAP, HR, rSO_2_, and HCT of the two groups (^#^*p* < 0.008) vs*.* t_1_ in **(a–c)** (^#^*p* < 0.017) vs*.* t_1_ in **(d)**.

The QoR-15 scores at different time points in the two groups are given in Table 2. The QoR-15 data did not violate sphericity, and the two-way repeated-measures analysis of variance for the QoR-15 data showed that the time effect, time × group effect, and group effect of the QoR-15 scores were significant in the two groups (*P* < 0.001, *P* = 0.007, and *P* = 0.001, respectively). The QoR-15 scores were low at 24 and 48 h after surgery in the C group (all *P* < 0.001) and at 24 h after surgery in the G group (*P* = 0.004). The QoR-15 scores at 24, 48, and 72 h after surgery were higher in the G group (*P* = 0.001, 0.001, and 0.005, respectively) than in the C group.

**Table 2:** The QoR-15 score at different time points in the two groups.

Time points	C group (*n* = 30)	G group (*n* = 30)	*t* values	*P*-values
The day before surgery	136.9 ± 5.1	137.4 ± 5.7	0.301	0.764
24 h after surgery	126.8 ± 5.4^a^	132.2 ± 6.0^a^^,^^b^	3.605	0.001
48 h after surgery	129.3 ± 6.4^a^	134.8 ± 5.8^b^	3.363	0.001
72 h after surgery	134.9 ± 3.8	138.3 ± 4.9^b^	2.954	0.005
*F* values	23.499	6.661	—	—
*P*-values	<0.001	<0.001	—	—

C group, control group; G group, goal-directed fluid therapy group.

Values are expressed as mean ± SD and the number of patients. Differences in the QoR-15 score between the two groups were analyzed using repeated-measures analysis of variance and the SNK *post-hoc* test with Bonferroni correction.

a *P* < 0.008 vs*.* the day before surgery.

b *P* < 0.05 vs. the C group.

The intraoperative fluid infusion volume and dopamine dosage values are given in Table 3. The intraoperative infusion volumes of Ringer's solution and hydroxyethyl starch were lower in the G group than in the C group (all *P* < 0.001). The intraoperative dopamine dosage in the G group was higher (*P* = 0.007) than that in the C group.

**Table 3:** The volume of intraoperative fluid infusion and blood loss and the dopamine dosage.

Parameter	C group (*n* = 30)	G group (*n* = 30)	*t* values	*P*-values
Ringer's solution (mL)	780.0 ± 119.3	516.7 ± 84.4^a^	9.876	< 0.001
Hydroxyethyl starch (mL)	216.7 ± 63.4	155.0 ± 48.0^a^	4.247	< 0.001
Dopamine dosage (mg)	2.4 ± 0.9	3.0 ± 0.7^a^	2.779	0.007
Intraoperative blood loss (mL)	17.3 ± 6.7	19.1 ± 6.7	1.106	0.273

C group, control group; G group, goal-directed fluid therapy group.

Values are expressed as mean ± SD and the number of patients. Differences in the intraoperative infusion volume of Ringer's solution, hydroxyethyl starch, and dopamine dosage between the two groups were analyzed using an independent samples t-test.

a *P* < 0.05 vs*.* the C group.

The adverse effects are listed in Table 4. There was no difference between the two groups in terms of the incidence of hypotension, bradycardia, dizziness, nausea, and vomiting (*P* = 0.333, 0.640, 0.506, and 0.382, respectively). The total incidence of adverse effects in the G group was significantly lower (*P* = 0.020) than that in the C group.

**Table 4:** Adverse events.

Adverse event	C group (*n* = 30)	G group (*n* = 30)	*χ^2^* values	*P*-values
Hypotension, *n* (%)	8 (26.7)	4 (13.3)	1.667	0.333
Bradycardia, *n* (%)	3 (10)	2 (6.7)	0.218	0.640
Dizziness, *n* (%)	7 (23.3)	4 (13.3)	1.002	0.506
Nausea and vomiting, *n* (%)	10 (33.3)	6 (20)	1.364	0.382
Total adverse events, *n* (%)	21 (70.0)	12 (40.0)^a^	5.455	0.020

C group, control group; G group, goal-directed fluid therapy group.

Values are expressed as the number of patients. Differences in hypotension, bradycardia, dizziness, nausea and vomiting, and the total incidence of adverse effects between the two groups were analyzed using a chi-square test or Fisher's exact test.

a *P* < 0.05 vs*.* the C group.

## Discussion

In this study, we found that the plasma concentrations of ICAM-1, IL-1β, syndecan-1, TNF-α, and VCAM-1 were significantly higher at the end of surgery, and intraoperative goal-directed fluid therapy could attenuate the increase in the postoperative CAM-1, IL-1β, syndecan-1, TNF-α, and VCAM-1 plasma concentrations; improve HCT and QoR-15 scores after surgery; and reduce the total incidence of adverse effects.

Intraoperative fluid therapy can effectively maintain the circulating blood volume, ensure tissue perfusion, and preserve water–electrolyte and acid–base balance, which are crucial for perioperative safety and postoperative recovery. Syndecan-1 has been identified as a valuable clinical biomarker for glycocalyx degradation (7). A clinical study has found that increasing the volume of the infusion fluid can lead to an increase in the plasma syndecan-1 concentration (8). In our study, the intraoperative infusion volume of Ringer's solution and hydroxyethyl starch in the control group was significantly increased, accompanied by a higher plasma concentration of syndecan-1, whereas in the goal-directed fluid therapy group, the intraoperative infusion volume of Ringer's solution and hydroxyethyl starch was decreased with a lower plasma concentration of syndecan-1. This indicated that intraoperative infusion of a large volume of fluids can lead to an increase in plasma syndecan-1 concentration (9). GDFT is an individualized fluid strategy based on hemodynamic monitoring, which can avoid positive fluid balance (10). In this study, the intraoperative fluid infusion volume was lower with the goal-directed fluid therapy strategy, which was consistent with the results of the aforementioned study. Syndecan-1, ICAM-1, and VCAM-1 are endothelial-related biomarkers (11), and their elevated plasma concentrations suggest that endothelial glycocalyx is damaged (12, 13). The plasma concentrations of syndecan-1, ICAM-1, and VCAM-1 were attenuated with intraoperative goal-directed fluid therapy in the present study. This indicates that intraoperative goal-directed fluid therapy in older patients can minimize the degradation of the endothelial glycocalyx (14).

ICAM-1 and VCAM-1 are adhesion molecules expressed by endothelial cells, which are shielded when the endothelial glycocalyx is intact. They are exposed after glycocalyx damage, promoting leukocyte adhesion and inflammatory response (1, 15). TNF-α and IL-1β are the most important proinflammatory cytokines, which play a crucial role in the initiation and development of inflammation (16, 17). In this study, although there was no difference in the plasma concentration of TNF-α between the two groups, the elevated plasma concentrations of ICAM-1, VCAM-1, and IL-1β were attenuated with intraoperative goal-directed fluid therapy, demonstrating that intraoperative goal-directed fluid therapy in older patients can significantly reduce inflammation (18).

A previous study found that during fluid overload, there is an increase in tissue sodium level and a translocation of endotoxins through a congested bowel wall, which may induce inflammation (19). However, we did not measure plasma endotoxin levels and tissue sodium levels in this study, and the precise mechanism by which the intraoperative goal-directed fluid therapy reduces inflammation in older patients remains unclear.

The MAP values significantly decreased after anesthesia induction in our study, indicating that surgical patients often suffer from hypotension after general anesthesia, especially older patients (20). Although supplementing for the preoperative hypovolemia that is caused by preoperative fasting and abstaining from drinking can reduce the occurrence of postanesthesia induction hypotension, an excessive fluid infusion may lead to fluid overload (21). The dopamine dosage was high and the intraoperative fluid infusion volume was low in the goal-directed fluid therapy group compared with those in the control group, which showed that appropriate administration of vasoactive drugs can prevent intraoperative fluid overload (22).

A prior clinical study has found that reducing intravenous infusion while maintaining hemodynamic stability can effectively shorten the length of hospital stay and reduce the risk of postoperative atelectasis and pulmonary infection (23). In this study, although differences in the QoR-15 scores between the two groups at 24 and 48 h were statistically significant, the 95% confidence interval (CI) of the difference achieved the minimal clinically important difference but not the clinically important significance. However, the incidence of total postoperative adverse events decreased under goal-directed fluid therapy with lower intraoperative fluid infusion, which was consistent with the aforementioned study. Prerehydration of hydroxyethyl starch can effectively reduce the incidence of postinduction hypotension in patients undergoing gastrointestinal surgery (24). Although cases of peritrophic nephropathy–like lesions or acute renal failure have been reported and are believed to be related to the use of hydroxyethyl starch (HES) (25), studies have demonstrated that the administration of HES has no influence on the renal function in critically ill patients (26, 27). In this study, older patients with kidney disease were excluded; the average intraoperative infusion volume of hydroxyethyl starch was only 3.4 mL/kg, which was much lower than the maximum daily dose of 33 mL/kg (28); and no patients suffered from postoperative renal dysfunction and coagulation dysfunction.

Nonetheless, there are several limitations in this study. First, inflammation may lead to delayed wound healing, increased risk of postoperative complications, prolonged hospitalization, and even serious problems such as sepsis (29). Although we found that goal-directed fluid therapy during surgery may reduce inflammation in older patients, the precise mechanism is still unclear. Second, the incidence of hypotension and nausea and vomiting in the C group was almost twice that in the G group, but the difference was not statistically significant, which may be due to the small sample size of this study. In future, the effect of goal-directed fluid therapy on intraoperative and postoperative adverse events in older patients should be investigated in a clinical trial with a large sample size. Finally, dopamine has been proven to be an effective treatment for hypotension during anesthesia, and a clinical study confirmed that dopamine was more effective than ephedrine in persistently increasing blood pressure by improving cardiac output (30–33) Meanwhile, animal experiments have confirmed that dopamine increases lung liquid clearance during mechanical ventilation (34). Currently, norepinephrine is commonly used as a vasoactive drug in goal-directed fluid therapy (35). In future, we will observe the effect of using norepinephrine instead of dopamine as a vasoactive drug for goal-directed fluid therapy on postoperative recovery in older patients.

To conclude, intraoperative goal-directed fluid therapy in non-critically ill older patients may attenuate endothelial glycocalyx degradation and inflammation, improve postoperative HCT, and reduce the overall incidence of adverse events.

## Data Availability

The original contributions presented in the study are included in the article/Supplementary Material, further inquiries can be directed to the corresponding author.
